# Modification of a Marine Pine Kraft Lignin Sample by Enzymatic Treatment with a *Pycnoporus cinnabarinus* Laccase

**DOI:** 10.3390/molecules28124873

**Published:** 2023-06-20

**Authors:** Sona Malric-Garajova, Florian Fortuna, Florian Pion, Elise Martin, Adithya Raveendran Thottathil, Audrey Guillemain, Annick Doan, Anne Lomascolo, Craig B. Faulds, Stéphanie Baumberger, Laurence Foulon, Brigitte Chabbert, Hélène de Baynast, Pascal Dubessay, Fabrice Audonnet, Emmanuel Bertrand, Giuliano Sciara, Sandra Tapin-Lingua, Paul-Henri Ducrot, Philippe Michaud, Véronique Aguié-Béghin, Eric Record

**Affiliations:** 1 INRAE, Aix-Marseille Université, UMR1163 Biodiversité et Biotechnologie Fongiques, 13288 Marseille, France; sonag@centrum.sk (S.M.-G.); annick.doan@inrae.fr (A.D.); anne.lomascolo@univ-amu.fr (A.L.); craig.faulds@univ-amu.fr (C.B.F.); emmanuel.bertrand@univ-amu.fr (E.B.); giuliano.sciara@inrae.fr (G.S.); 2 Université de Reims-Champagne-Ardenne, INRAE, Fractionnement des Agro-Ressources et Environnement (FARE), UMR A 614, 51097 Reims, France; florian.fortuna@inrae.fr (F.F.); laurence.foulon@inrae.fr (L.F.); brigitte.chabbert@inrae.fr (B.C.); veronique.aguie@inrae.fr (V.A.-B.); 3 Université Paris-Saclay, INRAE, AgroParisTech, Institut Jean-Pierre Bourgin (IJPB), 78000 Versailles, France; florian.pion@inrae.fr (F.P.); adithya-raveendran.thottathil@inrae.fr (A.R.T.); stephanie.baumberger@inrae.fr (S.B.); paul-henri.ducrot@inrae.fr (P.-H.D.); 4 Université Clermont Auvergne, Clermont Auvergne INP, CNRS, Institut Pascal (IP), 63000 Clermont-Ferrand, France; elise.martin@uca.fr (E.M.); helene.de-baynast@uca.fr (H.d.B.); pascal.dubessay@uca.fr (P.D.); fabrice.audonnet@uca.fr (F.A.); philippe.michaud@uca.fr (P.M.); 5 FCBA, InTechFibres Division, Domaine Universitaire CS 90125, Cedex 9, 38044 Grenoble, France; audrey.guillemain@fcba.fr (A.G.); sandra.tapin-lingua@fcba.fr (S.T.-L.)

**Keywords:** *Pycnoporus cinnabarinus* laccase, kraft lignin, industrial lignins, depolymerization, polymerization

## Abstract

Here, we report work on developing an enzymatic process to improve the functionalities of industrial lignin. A kraft lignin sample prepared from marine pine was treated with the high-redox-potential laccase from the basidiomycete fungus *Pycnoporus cinnabarinus* at three different concentrations and pH conditions, and with and without the chemical mediator 1-hydroxybenzotriazole (HBT). Laccase activity was tested in the presence and absence of kraft lignin. The optimum pH of *Pci*Lac was initially 4.0 in the presence and absence of lignin, but at incubation times over 6 h, higher activities were found at pH 4.5 in the presence of lignin. Structural changes in lignin were investigated by Fourier-transform infrared spectroscopy (FTIR) with differential scanning calorimetry (DSC), and solvent-extractable fractions were analyzed using high-performance size-exclusion chromatography (HPSEC) and gas chromatography–mass spectrometry (GC–MS). The FTIR spectral data were analyzed with two successive multivariate series using principal component analysis (PCA) and ANOVA statistical analysis to identify the best conditions for the largest range of chemical modifications. DSC combined with modulated DSC (MDSC) revealed that the greatest effect on glass transition temperature (Tg) was obtained at 130 U g cm^−1^ and pH 4.5, with the laccase alone or combined with HBT. HPSEC data suggested that the laccase treatments led to concomitant phenomena of oligomerization and depolymerization, and GC–MS revealed that the reactivity of the extractable phenolic monomers depended on the conditions tested. This study demonstrates that *P. cinnabarinus* laccase can be used to modify marine pine kraft lignin, and that the set of analytical methods implemented here provides a valuable tool for screening enzymatic treatment conditions.

## 1. Introduction

Wood is a renewable lignocellulosic raw material that is hugely valuable for producing fuels, materials and chemicals [1,2]. It is a composite material composed mainly of cellulose, hemicelluloses and lignin, together with extractable compounds [3,4]. Lignin is a naturally abundant branched aromatic polymer with UV absorption ability, antimicrobial, antioxidant, hydrophobic, amphiphilic, emulsifying and excellent binding properties [5,6,7]. Lignin interacts with cellulose and hemicelluloses via hydrogen bonds, van der Waals forces and covalent linkages in the cell walls of vascular plants [8,9]. The hydrophobic polymer is composed of cross-linked phenylpropanoid units derived from *p*-coumaryl alcohol, coniferyl alcohol and sinapyl alcohol, corresponding to hydroxyphenyl (H), guaiacyl (G) and syringyl (S) units, respectively. The major chemical functional groups in lignin are hydroxyl, methoxyl, carbonyl and carboxyl groups. Lignin can be extracted from wood through various different chemical pulping processes. The technical lignins coming from these processes fall into two main categories: sulfur-containing lignins (mainly kraft lignin found in the black liquor of the kraft pulping process and lignosulfonates from the liquor of the sulfite pulping process), and sulfur-free lignins (such as soda, organosolv and steam-explosion lignins) [10,11].

Kraft pulping is the dominant delignification process used to isolate and purify cellulose for the paper industry, which accounts for 85% of the total lignin produced worldwide [12]. Kraft pulping involves the treatment of wood chips at elevated temperatures of 140–170 °C with an alkaline aqueous solution of pulping chemicals [13]. The alkaline solution consists of an aqueous mixture of sodium hydroxide and sodium sulfide, so-called white liquor, and is used to cleave the bonds that link hemicellulose, cellulose and lignin. The main reaction in the kraft process is cleavage of lignin β-aryl ether linkages into alkali-soluble fragments. Isolated Kraft lignin contains many inter-unit linkages compared to native lignin. This technical lignin is considered to be more condensed than native lignin, at least because of the accumulation of 5-5′ and 4-O-5′ structures [14]. In addition, the phenolic hydroxyl content of dissolved kraft lignin is much higher than that of wood lignin [13]. However, its solubility in most common solvents and water is very low, except in highly alkaline environments (pH > 11) [15].

Laccases (Lacs) are multicopper enzymes that catalyze the oxidation of phenol to phenoxy radicals with the concomitant reduction of O_2_ to water [16]. Unlike many other enzymes, laccases have a wide range of substrates, including various types of phenols and polyphenols (including lignin), polyamines, aryl diamines and some inorganic ions. In addition, it has been shown that low-molecular-weight mediators, such as 1-hydroxy-benzo-triazole (HBT), can boost lignin oxidation at a distance from the enzyme active site and react with parts of the lignin polymer not accessible to Lacs enzyme alone [16]. Due to the complex structure of lignin, reaction mechanisms at the molecular level have mainly been studied using small model compounds representing structurally diverse lignins, and the reactions of different lignin model compounds in the presence and absence of mediators have been reviewed [17,18]. There are some reports on laccase-catalyzed modification of different lignins, but only a few studies have focused on laccase-mediated catalysis of technical lignins such as lignosulfonates and organosolv lignins [19,20,21,22]. Therefore, a better understanding of the enzymatic modification pathway of kraft lignin may contribute to the development of a milder, cleaner and safer process route for the formulation of new alternative bio-based materials implemented on the basis of enzymatically modified lignins.

The objective of this study was to explore the capacity of the laccase from the white-rot fungus *Pycnoporus cinnabarinus* (*Pci*Lac) to modify the structure of marine pine kraft lignin. The rationale for choosing this enzyme was that it is a high-redox-potential fungal enzyme that has previously been shown to possess a remarkable capacity to bleach softwood kraft pulp while preserving key pulp properties, but its biochemical behavior in the presence of a technical lignin and the physical–chemical modifications of the enzymatically modified lignins have not yet been characterized in detail [23]. Here, we implemented a multi-disciplinary approach including enzyme biochemistry and physico-chemical analysis in an effort to gain new and deeper insight into the mechanisms of lignin modification and deconstruction by a high-redox-active fungal laccase.

## 2. Results and Discussion

### 2.1. Biochemical Characterization of PciLac on Kraft Lignin

The pH of the kraft lignin suspension in water was about 2.7. This pH value is not favorable for *Pci*Lac activity, which has an optimum pH in the range pH 4–5 [24,25]. Moreover, kraft lignin is almost water-insoluble at acidic pH, which is likely to limit the effect of the enzyme performance on the substrate. In order to prepare the kraft lignin at a more favorable pH for *Pci*Lac activity, lignin was conditioned at varying pH levels from 4 to 7, and its solubility was assessed in these conditions. After centrifugation, visual observation of the supernatants revealed a slightly yellowish color at pH 4.5 and a deeper yellow color above pH 5. The supernatants showed peak adsorption at 280 nm, suggesting that lignin solubilization occurs above pH 4.5 under the tested conditions. The increase in supernatant color was estimated using gravimetric analysis of the lignin sediment and supernatant, which indicated a lignin solubility of 6% at pH 4.5 compared to 45% when assayed at pH 7 (Table 1).

**Figure 1:**
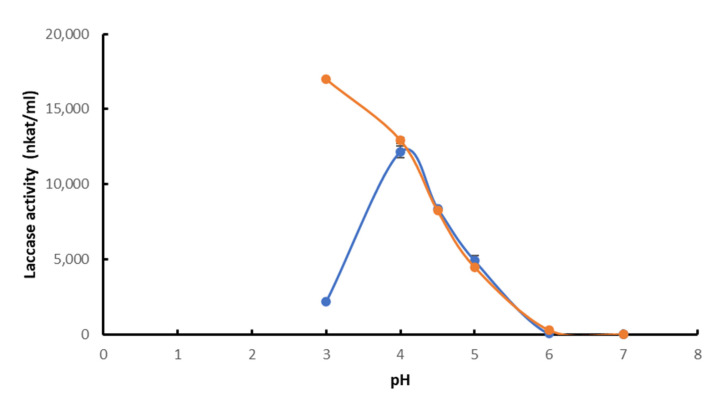
Optimal pH of *Pci*Lac in presence (blue) and absence (orange) of kraft lignin. Each datapoint (mean ± standard deviation) is the result of triplicate experiments.

Regarding enzyme performance, the optimum pH of *Pci*Lac was assayed at pH 4.0 in the presence of the kraft lignin. However, there was a clear difference at pH 3.0, at which laccase activity was higher in the absence of kraft lignin, whereas there was no activity in the presence of lignin. As previously observed, in the absence of lignin, ABTS activity increased when pH decreased, suggesting a faster oxidation of ABTS to the corresponding radical cation ABTS^+^ at pH 3.0 [24]. In the presence of lignin, the radical cation is likely reduced in the presence of phenolic compounds from the kraft lignin, resulting in competition with the ABTS oxidation by laccase, which visibly leads to a decrease in laccase activity. At pH 5 and in both the presence and absence of kraft lignin, *Pci*Lac showed a roughly 80–90% loss of its activity compared to measurements at pH 4.0 (Figure 1). There was no measurable laccase activity at pH 6.0 and above.

**Figure 2:**
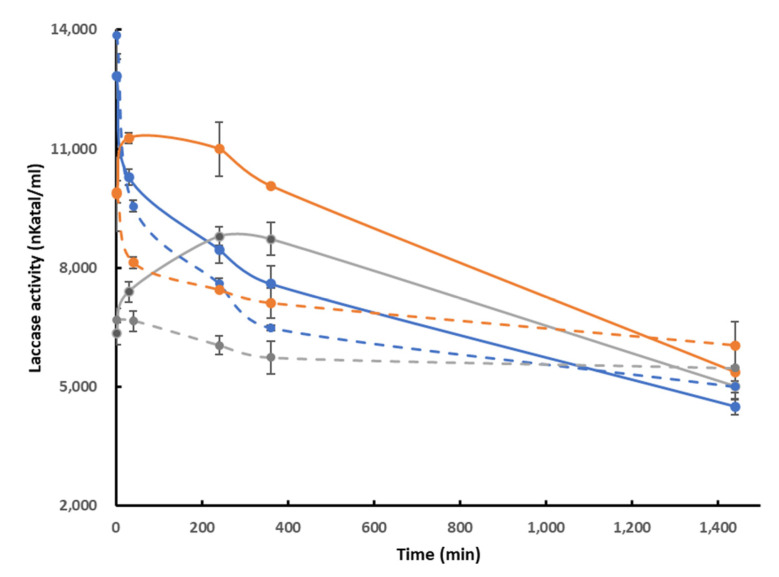
Time-course plot of the pH stability of laccase at pH 4.0 (blue), 4.5 (orange) and 5.0 (grey) in the presence (full line) and absence (interrupted line) of kraft lignin. Each datapoint (mean ± standard deviation) is the result of triplicate experiments.

In addition, the pH stability of the laccase in the presence of lignin was determined in the range pH 4 to 5 (Figure 2). At pH 4.0, which corresponded to the optimum pH of the laccase on ABTS, the presence of lignin had almost no effect on pH stability, whereas at pH 4.5 and 5.0, *Pci*Lac showed improved pH stability in the presence of kraft lignin during the first 240 min of incubation. Figure 2 also shows that, after 360 min of incubation, *Pci*Lac activity was higher at pH 4.5 than at pH 4 in both presence and absence of kraft lignin, and was 30% higher at pH 4.5 than at pH 4 in the presence of lignin. This activity difference remained at 10% at pH 5, probably because pH 5 is already unfavorable for ABTS oxidation. This result suggests that even a small increase in lignin solubility has a significant effect on enzyme performance.

### 2.2. Tracking Lignin Chemical Modification by Infrared Analysis

Kraft lignin conditioned at pH 4.5, pH 5.0 and pH 6.0 was modified by *Pci*Lac and the laccase mediator system (LMS) using HBT as a mediator. A control was used without laccase. Lignin was enzymatically treated at different pH levels (pH 4.5 and pH 5.0, with pH 6.0 as control) in the presence and absence of the enzyme (0 and 13 U g^−1^ lignin) and at two incubation times (30 min and 2 h).

**Figure 3:**
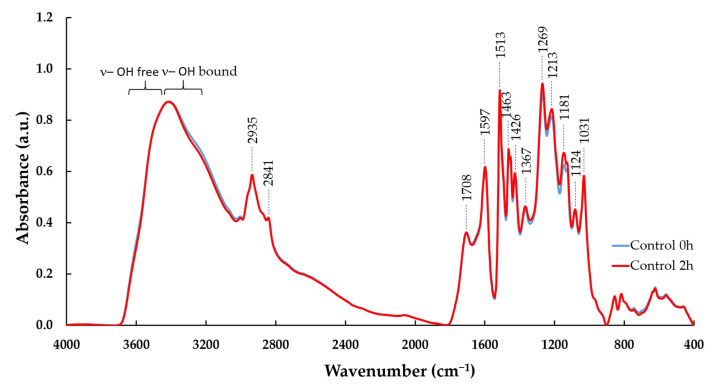
FTIR spectra of control kraft lignin incubated in pH 4.5 buffer without HBT for 0 and 2 h. Vertical lines indicate the main bands of lignin detailed in Table 1.

FTIR spectra (Figure 3) show the main absorption bands of kraft lignin [26,27,28]. These bands, detailed in Table 2, were assigned to the C-H stretching of the methoxyl groups (2873 cm^−1^, 2840 cm^−1^, 1463 cm^−1^, 1452 cm^−1^), carbonyl groups (C=O) in unconjugated ketones, carbonyl groups in esters, conjugated aldehydes and carboxylic acids (1708 cm^−1^), aromatic C–C double bonds (1598 cm^−1^, 1514 cm^−1^, 1031 cm^−1^), G ring (1269 cm^−1^, 1146 cm^−1^) and C-C and C-O bonds (1216 cm^−1^) according to the published literature [26]. S-type lignins usually show proportionally stronger signals at 1600 cm^−1^, whereas G-type lignins show proportionally stronger signals at 1510 cm^−1^, as was the case with the pine kraft lignin here (Figure 3). Control experiments with kraft lignin only (without enzyme) illustrated no substantial effect on FTIR spectra after dissolution in the buffer at pH 4.5, 5.0 and 6.0 without laccase and HBT nor after incubation of lignin at 30 °C for 2 h (Figure 3 and Supplementary Figure S1 and Supplementary Figure S2).

**Figure 4:**
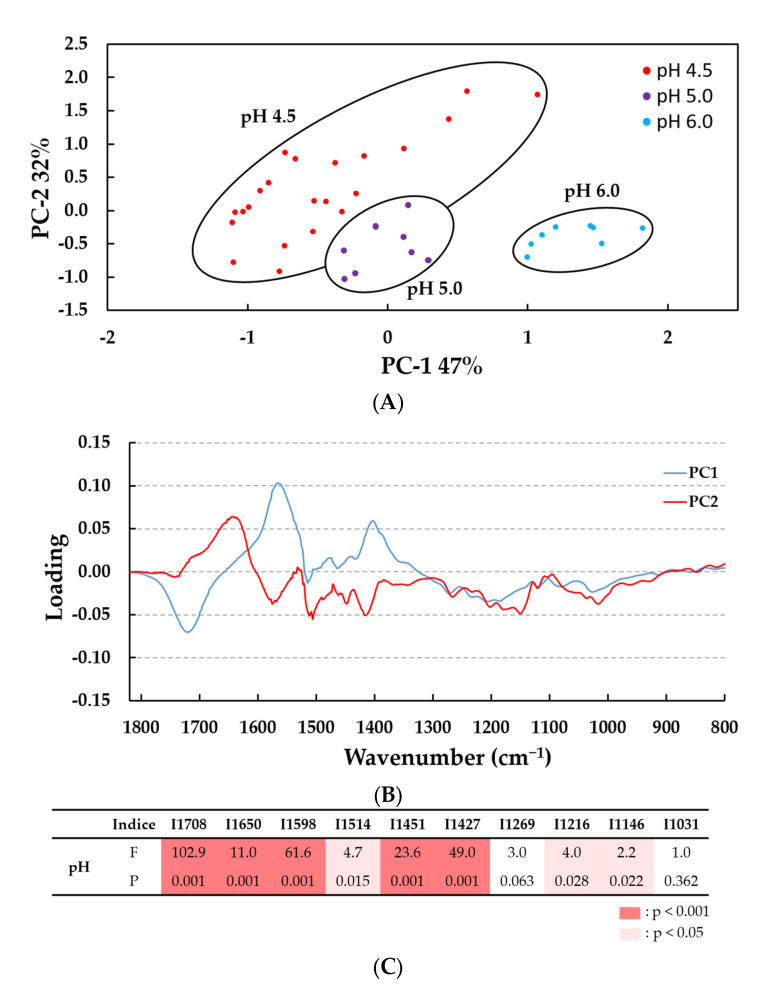
PCA analysis of the FTIR spectra in the 1800–700 cm^−1^ wavenumber range. (**A**) Score plot of PC2 (32%) vs. PC1(47%); (**B**) loadings profile highlighting the bands that contributed most to the separation of lignin samples (n = 38 samples). (**C**) Results of one-way ANOVA on 10 bands identified in the loadings profile. Laccase activated lignins at pH 4.5, 5.0 and 6.0 with 0 and 13 U g^−1^ lignin, with and without HBT, treated for 0, 0.5 and 2 h. Differences between pH levels of enzymatic treatment are significant at F statistic > 1 and *p* < 0.001 (in red) or *p* < 0.05 (in pink). The F test statistic is the ratio of two variances (variance between groups on variance within groups) and *p* value is the probability that determines if the difference between group means is statistically significant at 99.9% or 95%.

FTIR spectra were first analyzed for lignin treated with 13 U *Pci*Lac or without laccase (control) at the three pH values (pH 4.5, pH 5.0 and pH 6.0), with or without HBT (2%), and for either 30 min or 2 h of incubation. These spectral data (n = 38 spectra) were then subjected to a multivariate analysis employing qualitative principal component analysis (PCA) to identify differences between samples related to their evolution after the laccase treatment as a function of pH level (Figure 4). PCA analysis showed that the enzyme treatment revealed the greatest variations between IR spectra according to the pH value used. Indeed, the score plot on the plane of the first (PC1) and second (PC2) principal components, which together accounted for 79% of the total variance (Figure 4A), gives the plot of loadings using PC1 (47%) and PC2 (32%) versus the wavenumber variable (Figure 4B). As observed in the score plot, samples treated at pH 6.0 were clearly separated from samples treated at pH 5.0 and 4.5. This observation was in agreement with previous data showing the optimum pH of *Pci*Lac (Figure 1). Moreover, samples treated at pH 4.5 showed the highest variability between spectra. The main wavenumbers identified in the corresponding loading were in the range of IR bands from 1708 cm^−1^ to 1427 cm^−1^ (Figure 4B). We tested these specific bands with one-way ANOVA and found that eight bands varied significantly in intensity (five bands at *p* < 0.001 and three bands at *p* < 0.05) according to the pH variable, especially at pH 4.5: 1708 cm^−1^, 1650 cm^−1^ (C=O), 1598 cm^−1^ (C=C), 1451 cm^−1^ (C-H from methoxyl groups) and 1427 cm^−1^ (C-H from aromatic skeletal).

**Figure 5:**
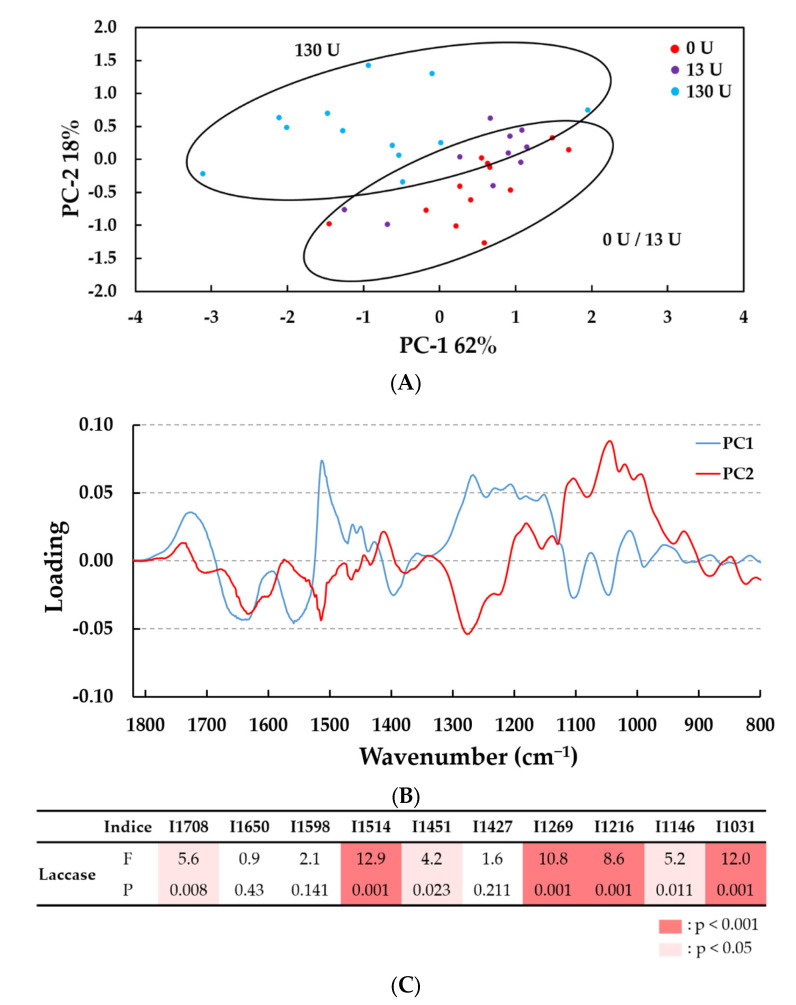
PCA analysis of the FTIR spectra in the 1800–700 cm^−1^ wavenumber range. (**A**) Score plot of PC2 (18%) vs. PC1 (62%); (**B**) loadings profile highlighting the bands that contributed most to the separation of lignin samples (n = 34 samples). (**C**) Results of one-way ANOVA on ten bands identified on the loadings profile. Laccase activated lignins at pH 4.5 with 0, 13 and 130 U/g lignin, with and without HBT, treated for 0, 0.5 and 2 h. Differences between concentration levels of enzymatic treatment are significant at F statistic > 1 and *p* < 0.001 (in red) or *p* < 0.05 (in pink). The F test statistic is the ratio of two variances (variance between groups on variance within groups) and the *p* value is the probability that determine if the difference between group means is statistically significant at 99.9% or 95%.

We therefore concluded that treating at pH 4.5 had the greatest effect on lignin. We then performed a second multivariate analysis between samples treated at pH 4.5 at three enzyme concentrations (0, 13 and 130 U *Pci*Lac g^−1^ lignin, with or without HBT; n = 34 spectra; Figure 5). PCA analysis showed that the enzyme treatment at 130 U g^−1^ led to significant differences to enzyme treatment at the other enzyme concentrations (0 and 13 U g^−1^). The score plot on the plane of the PC1and PC2, which together accounted for 80% of the total variance (Figure 5A), shows the loadings plot using PC1 (62%) and PC2 (18%) versus the wavenumber variable (Figure 5B).

The principal wavenumbers identified in the corresponding loading profiles were in the larger IR band range from 1708 cm^−1^ to 1031 cm^−1^ (Figure 5B). The one-way ANOVA showed significant differences in the intensity of seven bands according to laccase concentration (1514 cm^−1^, 1269 cm^−1^, 1216 cm^−1^ and 1031 cm^−1^ (*p* < 0.001) and 1708 cm^−1^, 1451 cm^−1^ and 1146 cm^−1^ (*p* < 0.05) (Figure 5C). Even though the FTIR spectra contained some differences that were not easy to distinguish visually (Supplementary Figure S3), PCA and ANOVA allowed us to conclude that laccase treatment generated the biggest chemical modifications in the lignin at pH 4.5 and 130 U g^−1^. The addition of HBT addition (2%) did not generate a significant effect in comparison with each respective control, except on the band at 1451 cm-1 (C-H bending from the methoxyl group, *p* < 0.05) (Supplementary Figure S4).

Thus, the decrease in the mean-intensity bands at 1708 (for carbonyl groups stretching in unconjugated ketones, carboxylic acids and ester groups), 1515 (for C=C aromatic skeletal), 1269 (for C–O stretching in the lignin and C–O linkage in guaiacyl aromatic methoxyl groups [30], 1216 and 1146 cm^−1^ was visible after 2 h of laccase treatment with or without HBT (Table 2, Figure S3 and Figure S4). All these variations could be assigned to a few modifications in the lignin aromatic structure. Notably, the relative decrease in intensity of the band at 1515 cm^−1^ (attributed to aromatic structure) associated with unchanged intensity of the band at 1598 cm^−1^ (attributed to condensed G [22]), suggests the modification of the interactions between lignin G units. Furthermore, small decreases in the bands at 1451 cm^−1^ (for C-H in methoxyl groups) and 1427 cm^−1^ (for aromatic skeletal vibrations combined with methoxyl plane deformation) support the hypothesis of a partial demethylation reaction by 130 U g^−1^ laccase at pH 4.5, in the presence of 2% HBT. Nevertheless, no major effect was observed near the phenolic groups at 1367 cm^−1^ (Figure S3). This supposes that this partial demethylation is simultaneously followed by an oxidation of phenol groups into carbonyl as shown by the shoulder increase at 1650 cm^−1^ corresponding to C=O stretching in conjugated *p*-substituted aryl ketones, in accordance with [22]. Finally, the increase in bands at 1031 cm^−1^ corresponding to alcohol groups (secondary and primary alcohol groups, respectively) could be related to the increase in low molecular compounds released in the reactional media after laccase treatment.

Thus, IR data seems to indicate more structural modification and less depolymerization in lignin treated this way [24,30]. The following HP-SEC analysis of kraft lignin and GC–MS analysis of extractible compounds will give more information of laccase treatment effects.

### 2.3. Analysis of Lignin Changes by a DSC/MDSC Method

As the composition and structure of the lignin can change in relation to glass transition temperature (T_g_) [31,32], a DSC analysis was set up on laccase-treated lignin samples (Table 3). T_g_ corresponds to the temperature range in which a reversible phenomenon of change in free volume and molecular mobility in the amorphous phase of a material takes place. It is a change from a hard solid to a more rubbery and malleable form. In polymers, there is a relationship between molecular weight and glass transition temperature that is usually described by the Fox–Flory equation [33]. Qualitatively, the observed decrease in T_g_ values is therefore correlated with the decrease in molecular weight. Indeed, shorter chains are more mobile and may act as plasticizers. As a result, less energy is needed to reach the glass transition, and it occurs at lower temperatures. Considering the results of our previous experiments, we only measured T_g_ at pH 4.5 as a function of laccase concentration and time of incubation at this pH value. Control experiments (BB48, without enzyme and HBT and 55-3, without laccase) showed a T_g_ value of around 188–190 °C, which demonstrates that the HBT alone had no effect on this T_g_ (Table 3).

All samples treated with *Pci*Lac alone showed a significant decrease in T_g_, ranging from −6.0 °C (BB50) to −19.1 °C (BB77-1), compared to untreated kraft lignin (control BB48). In addition, when the enzyme was used alone for lignin treatment, incubation time had no significant effect on T_g_, whereas a larger amount of enzyme led to a lower T_g_. For example, the samples treated at pH 4.5 with 13 U g cm^−1^ of laccase for 0.5 h or 2 h had a relatively similar T_g_ of 181.8 °C and 184.3 °C, respectively. Similarly, samples treated with 130 U *Pci*Lac for incubation times of 0.5 and 2 h had a relatively similar T_g_ of 173.6 °C and 171.2 °C, respectively. However, when increasing the amount of laccase (13 against 130 U), larger decreases in T_g_ were found, with the lowest T_g_ found with sample BB77-1 (130 U, 2 h) with a value of 171.2 °C, which corresponds to a loss of 19.1 °C compared to the control BB48. As a first hypothesis, we assume that a larger amount of laccase might lead to the greatest change in the lignin, and generate lower molecular weights. A second set of assays were run, adding the synthetic mediator HBT. This LMS led to lower T_g_ values than treatments with *Pci*Lac1 alone, confirming that the HBT mediator had an effect that potentiated the laccase activity on lignin. At low laccase activity (13 U), there was no difference between tests with or without HBT (181.8 °C vs. 181.6 °C, respectively) even after a longer incubation time (2 h) (184.3 °C vs. 186.1 °C, respectively). For 130 U *Pci*Lac, a short incubation time (0.5 h) led to only a 3 °C decrease, whereas a 2 h incubation time led to a significantly higher decrease of 4.6 °C between samples treated with or without HBT. As a result, T_g_ reached 166.6 °C for a combined laccase/HBT treatment, i.e., a decrease of 15.5 °C compared to the control with HBT (BB50).

We therefore concluded that the treatment conditions at pH 4.5 with *Pci*Lac alone at 130 U g cm-1 and the laccase/HBT combination generated the greatest change in the kraft lignin. The lowest T_g_ (166.6 °C) measured was for the lignin treated at pH 4.5 with 130 U g cm^−1^ of laccase and 2% (*w*/*w* lignin) of HBT for 2 h, demonstrating that the synthetic mediator potentiates the effect of the *Pci*Lac. A Pearson correlation analysis between T_g_ values and the intensity of the seven discriminant FTIR bands (Figure 5C) showed positive relationships between T_g_ and the intensity of four bands, i.e., 1708, 1515 and 1269 cm^−1^ with a correlation coefficient R > 0.87 (*p* < 0.001) and 1216 cm^−1^ with R = 0.806 (*p* = 0.005; Figure S5). These results suggest that the decrease in T_g_ could be correlated to the decrease in carbonyl groups and some C=C bands of G rings.

### 2.4. Lignin Analysis by HPSEC

**Figure 6:**
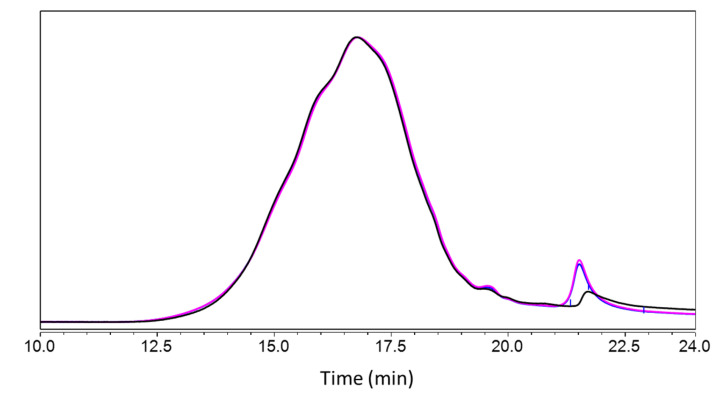
Size exclusion chromatograms after acetylation of control kraft lignin (black line, untreated lignin), BB48 (blue line, at pH 4.5, then directly freeze-dried) and BB49 (pink line, dissolved at pH 4.5, kept for 2 h, then freeze-dried). The chromatograms were normalized on the full range.

HPSEC of the kraft lignin in THF confirmed a polymeric distribution between 80 and 21,240 g·mol^−1^ (Mn 776, Mw 2660 and P.D.I 3.43) of macromolecules absorbing UV at 280 nm (Figure 6), plus minor amounts of low-molar-mass compounds.

Comparison of kraft lignin prepared in 10 mM sodium acetate buffer (BB48 and BB49) vs. control found a slight change in higher-molecular-mass components and an emergence of lower-molecular-mass components at around 19.5 min, but the general appearance of the chromatograms was similar overall. This indicates that adding the kraft lignin to the buffer for 2 h followed by freeze-drying treatment had no effect on the lignin.

**Figure 7:**
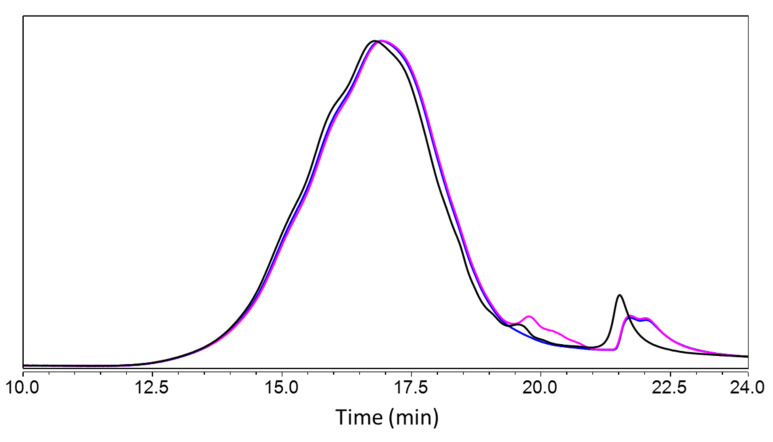
Size exclusion chromatograms after acetylation of samples treated at pH 4.5: BB49 (black line, kraft lignin dissolved in 10 mM sodium acetate buffer and kept for 2 h without enzyme, then freeze-dried), BB77 (blue lignin, kraft lignin incubated for 2 h in presence of laccase at 130 U g^−1^, then freeze-dried) and BB78 (pink line, kraft lignin incubated for 2 h in presence of laccase at 130 U g^−1^ and 2% HBT as mediator, then freeze-dried). The chromatograms were normalized on the full range.

Comparison of the size-exclusion chromatograms of laccase-treated samples (BB77 and BB78) vs. control (BB49) found an increase in higher-molar-mass components (rt 11.5–16.5 min), suggesting a condensation of the lignin, and an emergence of lower-molecular-mass components at around 19.5 min, presumably corresponding to the depolymerization of the lignin (Figure 7). These combined observations pointed to simultaneous phenomena of oligomerization and depolymerization due to a potential ambivalence of laccase activity. This phenomenon has been described for the treatment of different lignins with laccases from different sources, i.e., bacteria and fungi [34]. In this work, laccases were shown to oxidize and depolymerize phenolic lignin and, simultaneously, the phenoxy radicals formed by the action of laccases on the phenolic units and by the cleavage of inter-unit linkages of the lignin led to lignin condensation caused by radical–radical coupling. Furthermore, the shape of the chromatogram showed further changes related to the laccase treatment, such as slight shoulders on the main peak (at about 15, 16 and 17 min) in the kraft lignin control, whereas the shape was Gaussian after laccase treatment in the presence and absence of HBT, which is probably linked to this dual phenomenon. The peak at 21.5 min signals a toluene residue from the acetylation process and does not warrant further analysis.

### 2.5. Further Exploration of the Extractable Compounds through GC–MS Analysis

In order to detect possible changes in the composition of the low-molar-mass fraction, the extractable compounds present in the lignin samples were analyzed with GC–MS after silylation. The method used was suitable for the detection of compounds with masses equivalent to lignin monomers and dimers (*m*/*z* in the 35–500 mass unit range). The phenolic compounds detected showed guaiacyl structures, with the main compounds being guaiacol, vanillin, acetovanillone, homovanillyl alcohol, vanillic acid and vanilpropanol. In addition, we also found pine-specific extractable compounds such as dehydroabietic and pimaric acids, which are expected in this kind of pine kraft lignin [35]. Comparison of the treated samples against the corresponding controls (lignins incubated in the same conditions, but in absence of laccase and HBT) led to the two following conclusions: (i) no new extractive compounds appeared to be released and the concentration of the identified extractive compounds did not increase and therefore did not indicate end-wise depolymerization; (ii) in some treatments, the content of extractable compounds decreased relative to the corresponding control (Table 4), suggesting that in these conditions they were oxidized by *Pci*Lac and they reacted by oligomerization or were coupled to the lignin polymer, as described by Ibarra et al. [34].

The compounds most affected were homovanillyl alcohol and vanilpropanol, with more than 90% conversion whatever the laccase treatment conditions. Vanillin and acetovanillone were also converted via this treatment, but their loss was higher at the higher laccase concentration (130 U g^−1^ instead of 13U), indicating that mechanisms catalyzed by laccase oxidation took place. Acetovanillone and vanillin consumption was also higher in the presence of HBT, with decreases of about 40% and 60%, respectively. These two compounds showed the greatest change at pH 4.5, with 130 U g^−1^ laccase and 2% HBT, which are the conditions that also showed substantial variations in the FT-IR and Tg analyses. For vanillic acid, we observed the same loss when increasing the laccase concentration, but the addition of HBT had no further effect. These results suggested that laccase was able to catalyze the oxidation of kraft lignin phenolic extractable compounds, which could explain their subsequent oligomerization and/or coupling to the lignin revealed by HPSEC.

## 3. Material and Methods

### 3.1. Preparation of Lignin

Kraft lignin from maritime pine (*Pinus maritima*) was prepared by FCBA (Forêt Cellulose Bois-construction Ameublement, Champs-sur-Marne, France). Briefly, kraft pulp was resuspended in water or in a solution of HBT at about 10% consistency and adjusted to the desired pH with 1M NaOH. pH was checked twice per day and re-adjusted if necessary until reaching a stable value. After this step, sodium acetate buffer (final concentration of 10 mM) was added to obtain a final concentration of 5% (*w*/*w*).

### 3.2. Production of PciLac

The high-redox-potential laccase *Pci*Lac was produced and purified as previously described [24,25]. Standard laccase activity was measured by monitoring the oxidation of 500 µmol·L^−1^ 2,2′-azino-bis(3-ethylbenzothiazoline-6-sulfonic acid (ABTS) at 420 nm (ε = 36,000 L·cm^−1^·mol^−1^) in tartrate buffer (50 mmol·L^−1^, pH 4, pH 4.5, or pH 5) at 25 °C for 1 min. Enzyme activity was expressed in units (U) as μmol of ABTS oxidized per minute.

### 3.3. Biochemical Properties of PciLac in Presence of Kraft Lignin

The optimum pH of the laccase in presence and absence of kraft lignin was measured at 30 °C in 10 mM citrate–phosphate buffer over a pH range of 3 to 7, in one-pH-unit increments. Likewise, pH stability was analyzed by incubating *Pci*Lac in the presence and absence of kraft lignin at 30 °C for 4, 30, 60, 120, 360 min and 24 h in 10 mM sodium acetate buffer at pH 4, pH 4.5 and pH 5.

### 3.4. Lignin Treatment with Laccase

One gram of lignin or lignin/HBT samples prepared at pH 4.5, pH 5.0 and pH 6.0 was treated with varying amounts of enzyme at 30 °C for 0.5 or 2 h. Enzymatic treatment was performed in aqueous medium with shaking, using a lignin concentration of 5.0% (*w*/*w*) and two different enzyme concentrations (13 and 130 U *Pci*Lac g^−1^ kraft lignin). Reaction pH was adjusted as described above. HBT was used at a concentration of 2.0% (*w*/*w* lignin). The final volume of the reaction was 20 mL. The reactions were stopped by freeze-drying. Controls were obtained by treating the lignin under the same reaction conditions but without addition of enzyme or the HBT mediator. All reactions were performed in duplicate at least.

### 3.5. Lignin Characterization by FTIR

Fourier-transform infrared spectroscopy (FTIR) spectra were recorded on potassium bromide (KBr) pellets using a Nicolet 6700 spectrophotometer. The pellets were prepared by mixing 200 mg of spectroscopy-grade KBr with 5 mg lignin. Spectra were recorded using 16 scans at a resolution of 4 cm^−1^ from 400 to 4000 cm^−1^ with background subtraction. Each lignin sample was scanned three times and baseline-corrected and normalized using the area of the spectra from 700 to 4000 cm^−1^. This spectral data was used as an input in qualitative principal component analysis (PCA) to provide scores (sample-related, evolution in pH, enzyme concentration, or time) and loading profiles (variable-related, highlighting the characteristic bands that contribute most to the separation of laccase-activated lignin) that quantify how much of each of the original variables served to define each principal component (PC) and best describe the sources of variance in the data. The score plots are the projections of the original data onto the new vector space, defined by the loadings. These plots reveal the sample groups and clusters. PCA analysis was performed using Unscrambler^®^ X chemometric software 10.2 (AspenTech, Bedford, MA, USA) and combined with quantitative statistical analysis (ANOVA) using SigmaPlot 11.0 (Systat Software, Chicago, IL, USA).

### 3.6. Characterization of the Enzymatically Modified Kraft Lignin by a DSC/MDSC Method

The glass transition temperature (Tg) of lignin was measured by both differential scanning calorimetry (DSC) and modulated differential scanning calorimetry (MDSC) using a Q2000 apparatus (TA Instruments, New Castle, DE, USA) equipped with an RCS cooling system (TA Instruments, New Castle, DE, USA). Data was recorded using a TA Instrument Explorer and analyzed using TA Universal Analysis software (TA Instruments, Guyancourt, France). Approximately 7 mg of each lignin sample was sealed in a hermetic aluminum crucible. Analyses were performed under a nitrogen atmosphere (technical N2, 25 mL·min^−1^). All DSC measurements were carried out via a two-step procedure: the first phase, which aims to erase the thermal history of the samples, is a standard DSC heat/cool cycle with a ramp of 10 °C min^−1^ over a temperature range of 0–250 °C; the second phase, which aims to assess the Tg of samples, is an MDSC heating ramp of 5 °C min^−1^ with an oscillation period of 60 s and an amplitude of ±0.796 °C over the same of 0–250 °C temperature range. In both phases, we used the parameters recommended by TA Instruments.

### 3.7. HP-SEC Analysis of Kraft Lignin

Ten mg of sample was weighed into a capped tube and then added with 250 µL of anhydrous pyridine. The mixture was cooled to 0 °C before adding 500 µL acetic anhydride, then stirred overnight on an orbital stirrer at room temperature. The mixture was then cooled to 0 °C before adding 1 mL of methanol. Excess reagent and solvents were removed by successive addition of toluene and methanol under a vacuum. The resulting samples were dissolved in 5 mL of tetrahydrofuran (THF) and filtered prior to analysis by HPSEC (0.45 µm GHP Acrodisc microfilters, Merck, Darmstadt, DE, USA). SEC analysis of acetylated samples was performed using THF stabilized with butylated hydroxytoluene (BHT) as eluent at 1 mL min^−1^ (Dionex Ultimate 3000 Pump, Thermo Fisher Scientific, Waltham, MA, USA). Then, 10 µL was injected (Dionex Ultimate 3000 Autosampler, Thermo Fisher Scientific, Waltham, MA, USA) on a Mixed C column (5 µm, 7.5 × 600 mm; Polymer Laboratories, Long Beach, CA, USA) and the signal was read at 280 nm (Dionex Ultimate 3000 UV/vis detector, Thermo Fisher Scientific, Waltham, MA, USA). Molar mass distributions were determined by a calibration curve based on polystyrene standards (Polymer Laboratories, Church Stretton, UK).

### 3.8. Analysis of Extractable Compounds by GC–MS

Thirty mg of lignin samples were shaken for 1 h in 2 mL of deionized water acidified with 0.1% formic acid, then added with 100 µL internal standard (0.5 mg mL^−1^ *ortho*-coumaric acid in acetonitrile and 2 mL ethyl acetate (EtOAc), and then vortexed for 30 s. After phase separation, 1 mL of the organic phase was collected and dried under nitrogen. Extractable compounds were resuspended in 250 µL EtOAc, and 20 µL of this solution previously dried with Na_2_SO_4_ was silylated in the presence of 10 µL pyridine and 100 µL of *N,O*-bis(trimethylsilyl)trifluoroacetamide (BSTFA) for 2 h at room temperature.

0.2 µL of the silylated extractable compounds was injected in splitless mode (7693A Autosampler, Agilent Technologies, Santa Clara, CA, USA) onto a CP8912 column (30 m × 0.25 mm × 0.25 µm, Agilent Technologies), and submitted to the following temperature program (with helium as carrier gas): hold 1 min at 45 °C, ramp at 30 °C/min until 110 °C, then ramp at 3 °C/min until 300 °C, hold for 10 min (8860 GC System, Agilent Technologies). The chromatographic system was combined with a quadrupole MS system (5977B MSD, Agilent Technologies, Santa Clara, CA, USA) operating with electron-impact ionization (70 eV) and positive-mode detection, with a source at 250 °C and a transfer line at 270 °C, over a scanning range of 50–650 *m*/*z*.

## 4. Conclusions

This study set out to compare different laccase treatments under different conditions for the enzymatic modification of marine pine kraft lignin and to select the best conditions for potential valorization in future experiments. The results showed that the laccase was more active in the presence of kraft lignin, which could stabilize its enzymatic activity. Several physical–chemical techniques were co-implemented and revealed that the process conditions of 130 U g^−1^ laccase at pH 4.5 with HBT led to the greatest range of chemical changes. These modifications concern a weak but significant decrease in seven specific band intensities assigned to carbonyl groups (C=O), aromatic rings (C=C) and methoxy groups (-C-O). This finding supports the hypothesis of partial depolymerization and demethylation in relation to the presence of lower-molar-mass extractible fractions of the kraft lignin. However, this depolymerization seems to be rapidly followed by uncontrolled recondensation phenomena. Complementary experiments are now needed to exploit these enzymatically modified lignins in practical applications, such as the production of adhesives. A more extensive depolymerization by using cooperative enzymes, such as glucose dehydrogenases [36,37], which act on the newly formed phenoxy radicals and control their concentration and reactivity through potential reduction, could be also achievable and warrants further exploration under the favorable conditions identified here.

## Figures and Tables

**Table 1:** Gravimetric determination of soluble and insoluble lignin content.

pH	Insoluble Lignin (% *w*/*w*)	Soluble Lignin (% *w*/*w*)
4.5	93.6	6.4
7	55.1	44.9
8	39.8	60.2
9	27.8	72.2
10	1.8	98.2

**Table 2:** Main FTIR absorption bands of lignin samples from [26,29].

Band Wavelength (cm^−1^)	Assigned Function
3600–3200	OH groups and hydrogen bonds
2934	Methyl groups, methoxyl C-H stretching
2873	Methoxyl C-H stretching
2840	Methoxyl C-H stretching
1708	C=O stretching in unconjugated ketones, carbonyls, and ester groups; conjugated aldehydes and carboxylic acids
1650	C=O stretching in conjugated p-substituted aryl ketone
1598	C=C aromatic skeletal, C=O stretching, G condensed > G etherified
1514	C=C aromatic skeletal
1463	C-H def. (deformation) in CH_3_ and CH_2_
1452	C-H bending from methoxyl group
1427	Aromatic skeletal combined with C-H def.
1367	aliphatic C-H stretching in CH3 (not in O-Me), phenolic group
1269	G ring; C=O stretching
1216	C-C, C-O, C=O stretching
1146	Aromatic C-H def., G units
1127	aromatic C-H def., C=O stretching
1081	C-O def. in secondary alcohols and aliphatic ethers
1031	Aromatic C–H in plane def., plus C–O def. in primary alcohols, C=O stretching (unconjugated)
854	C-H of G units

**Table 3:** T_g_ values of lignin samples treated at pH 4.5 for different time, enzyme activity, and mediator supplementation conditions.

Sample	Time (h)	Laccase (U g^−1^)	HBT (%)	Tg (°C)
FC1-BB48	0	0	0	190.3
FC1-BB55-3	0	0	2	188.0
FC1-BB50	0.5	13	0	181.8
FC1-BB57	0.5	13	2	181.6
FC1-BB51	2	13	0	184.3
FC1-BB58	2	13	2	186.1
FC1-BB54	0.5	130	0	173.6
FC1-BB60	0.5	130	2	170.6
FC1-BB77-1	2	130	0	171.2
FC1-BB78-1	2	130	2	166.6

**Table 4:** Extractive compounds estimated with GC–MS in relative extractability compared to the corresponding control. For each chromatogram, the area of the compound was divided by area of the internal standard and normalized to sample weight; the values thus obtained for treated samples were divided by the values of the corresponding controls to estimate the percentage of residual extractive compounds. V = vanillin, AV = acetovanillone, HV-OH = homovanillyl alcohol, VA = vanillic acid, VP-OH = vanilpropanol.

Samples/Corresponding Controls	Laccase (U g^−1^)	HBT (%)	V	AV	HV-OH	VA	VP-OH
FC1-BB51/BB49	13	0	90.0	65.5	4.7	70.8	15.2
FC1-BB77/BB49	130	0	31.0	25.2	9.7	53.7	15.4
FC1-BB58/BB56	13	2	70.0	55.8	8.1	42.8	14.5
FC1-BB78/BB56	130	2	20.1	17.2	5.6	45.7	12.8

## Data Availability

All data are available in the main text or the Supplementary Materials.

## Supplementary Materials

The following supporting information can be downloaded at: https://www.mdpi.com/article/10.3390/molecules28124873/s1; Figure S1: FTIR spectra of control kraft lignin incubated in buffer at pH 5.0 without HBT, for 0 and 2 h; Figure S2: FTIR spectra of control kraft lignin incubated in buffer at pH 6.0 without HBT, for 0 and 2 h; Figure S3: FTIR spectra of kraft lignin treated by *Pci*Lac with HBT (2%) for 0, 0.5 and 2 h. Insert: full range. The vertical lines indicate the main bands of lignin detailed in Table 2; Figure S4: (A) R values of 10 FTIR bands identified from loading profiles. R is the intensity ratio of each FTIR band between kraft lignin treated by *Pci*Lac (130 U/g for 2 h) with and without HBT (2%) and the respective control. (B) Results of two-way ANOVA on 10 bands identified from loading profiles. Laccase activated lignins at pH 4.5 with 0, 13 and 130 U/g of *Pci*Lac, with and without HBT for 0, 0.5 and 2 h. Differences vs. control were significant at F statistic > 1 and *p* < 0.001 (red) or *p* < 0.05 (pink); Figure S5: Scatter plot of T_g_ and the 7 significant FTIR bands (I1708, I1515, I1451, I1269, I1216, I1146, I1031) from Figure 5C. Highlighted squares were the pair of variables with a positive correlation coefficient R > 0.87 and *p* values below 0.001, except T_g_ & I1216 with R = 0.806 and *p* = 0.005.
