# Supplementary material for: Modification of a Marine Pine Kraft Lignin Sample by Enzymatic Treatment with a Pycnoporus cinnabarinus Laccase
Source: Molecules. 2023 Jun 20;28(12):4873. doi: 10.3390/molecules28124873 (PMC10305415; doi:10.3390/molecules28124873)
Supplement: Supplementary file 1 [file molecules-28-04873-s001.zip › molecules-2423033-supplementary.pdf]

Supplementary data

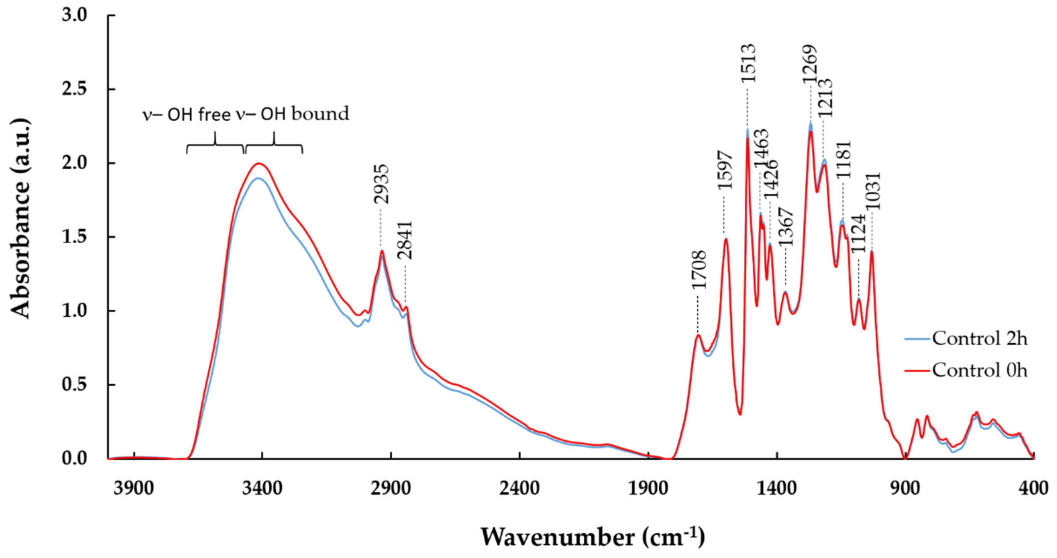

Figure S1: FTIR spectra of control kraft lignin incubated in buffer at pH 5.0 without HBT, for 0 and 2 hours.

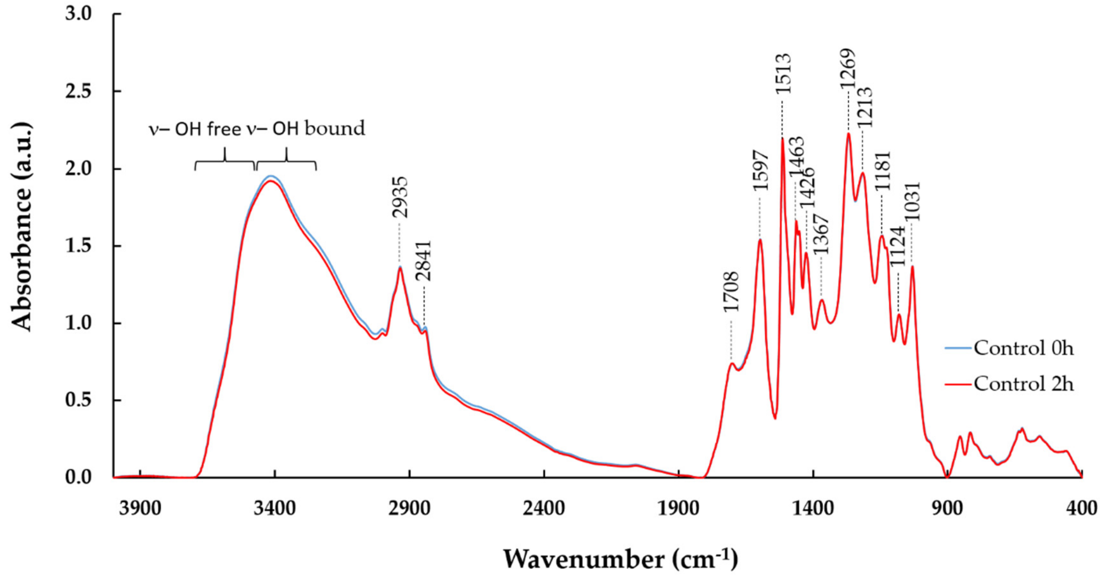

Figure S2: FTIR spectra of control kraft lignin incubated in buffer at pH 6.0 without HBT, for 0 and 2 hours.

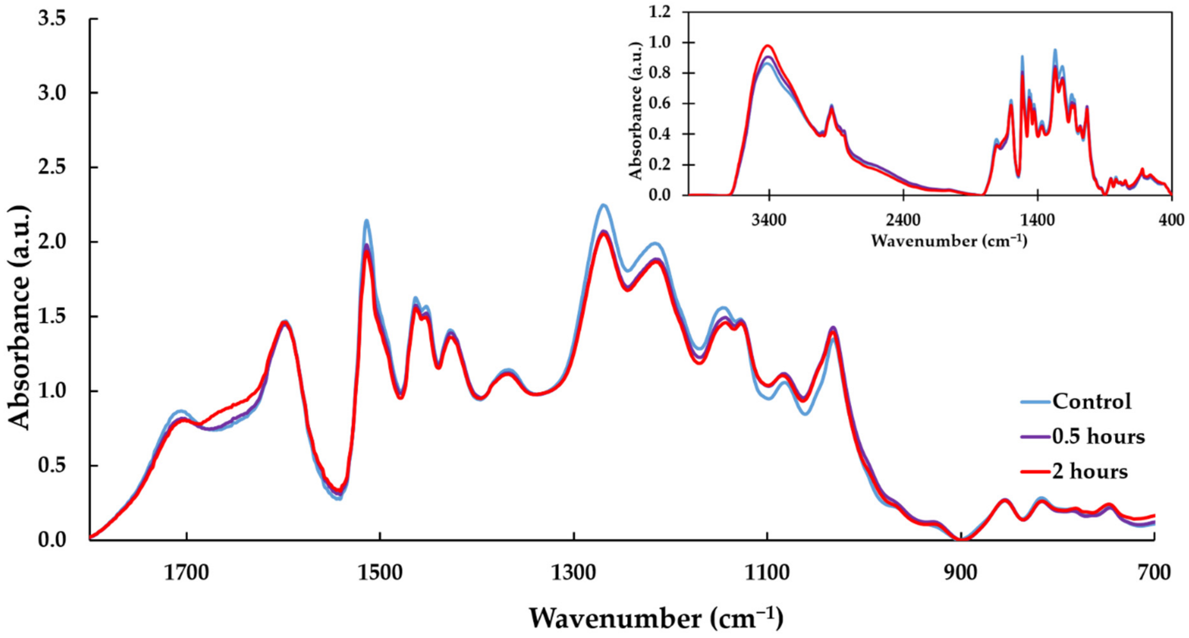

Figure S3: FTIR spectra of kraft lignin treated by *Pci*Lac with HBT (2%) for 0, 0.5 and 2 hours. Insert: full range. The vertical lines indicate the main bands of lignin detailed in Table 2.

A

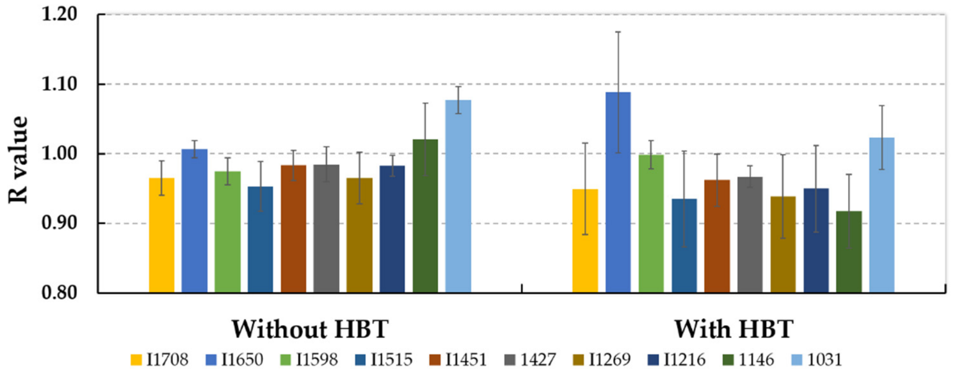

B

Two way ANOVA

	Indice	I1708	I1650	I1598	I1514	I1451	I1427	I1269	I1216	I1146	I1031
**Laccase**	F	5.2	0.9	2.2	13.4	5.3	1.7	11.5	8.9	5.1	12.9
	P	0.012	0.436	0.129	0.001	0.011	0.201	0.001	0.001	0.013	0.001
**HBT**	F	0.4	0.1	2.0	0.9	2.5	0.6	0.4	0.01	0.4	0.05
	P	0.555	0.754	0.165	0.344	0.125	0.450	0.511	0.933	0.539	0.825
**Laccase X HBT**	F	0.3	1.2	1.4	1.5	4.5	1.9	2.3	2.0	1.1	2.6
	P	0.765	0.304	0.273	0.236	0.021	0.163	0.121	0.151	0.359	0.091

: $p < 0.001$

: $p < 0.05$

Figure S4: (A) R values of 10 FTIR bands identified from loading profiles. R is the intensity ratio of each FTIR band between kraft lignin treated by *Pci*Lac (130 U/g for 2 hours) with and without HBT (2%) and the respective control. (B) Results of two-way ANOVA on 10 bands identified from loading profiles. Laccase activated lignins at pH 4.5 with 0, 13 and 130 U/g of *Pci*Lac, with and without HBT for 0, 0.5 and 2 hours. Differences vs. control were significant at F statistic > 1 and P<0.001 (red) or P<0.05 (pink).

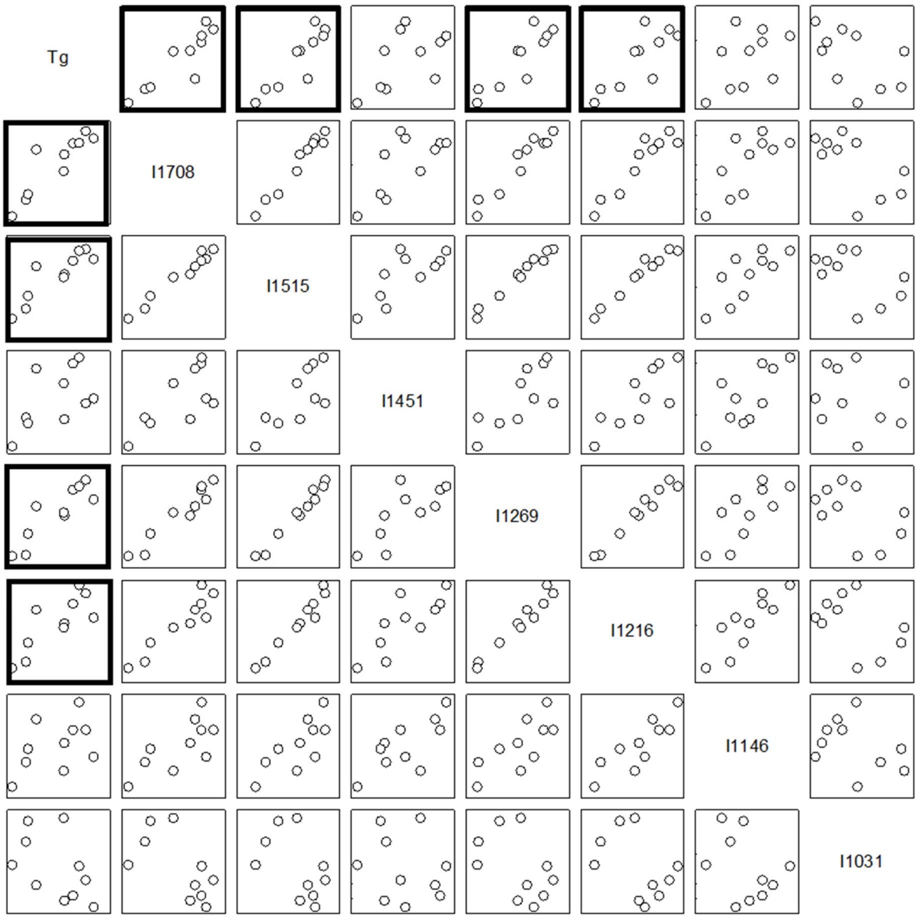

Figure S5: Scatter plot of Tg and the 7 significant FTIR bands (I1708, I1515, I1451, I1269, I1216, I1146, I1031) from figure 5c. Highlighted squares were the pair of variables with a positive correlation coefficient R > 0.87 and P values below 0.001, except Tg & I1216 with R = 0.806 and P = 0.005.
